# The impact of the advanced practice nursing role on quality of care, clinical outcomes, patient satisfaction, and cost in the emergency and critical care settings: a systematic review

**DOI:** 10.1186/s12960-017-0237-9

**Published:** 2017-09-11

**Authors:** Brigitte Fong Yeong Woo, Jasmine Xin Yu Lee, Wilson Wai San Tam

**Affiliations:** 1 Alice Lee Centre for Nursing Studies, Yong Loo Lin School of Medicine, National University of Singapore, Level 2, Clinical Research Centre, Block MD11, 10 Medical Drive, Singapore, 117597 Singapore; 2 National University Heart Centre Singapore, National University Hospital, 5 Lower Kent Ridge Road, Main Building 1, Level 2, Singapore, 119074 Singapore

## Abstract

**Background:**

The prevalence of chronic illness and multimorbidity rises with population aging, thereby increasing the acuity of care. Consequently, the demand for emergency and critical care services has increased. However, the forecasted requirements for physicians have shown a continued shortage. Among efforts underway to search for innovations to strengthen the workforce, there is a heightened interest to have nurses in advanced practice participate in patient care at a great extent. Therefore, it is of interest to evaluate the impact of increasing the autonomy of nurses assuming advanced practice roles in emergency and critical care settings on patient outcomes.

**Objectives:**

The objectives of this study are to present, critically appraise, and synthesize the best available evidence on the impact of advanced practice nursing on quality of care, clinical outcomes, patient satisfaction, and cost in emergency and critical care settings.

**Review methods:**

A comprehensive and systematic search of nine electronic databases and a hand-search of two key journals from 2006 to 2016 were conducted to identify studies evaluating the impact of advanced practice nursing in the emergency and critical care settings. Two authors were involved selecting the studies based on the inclusion criteria. Out of the original search yield of 12,061 studies, 15 studies were chosen for appraisal of methodological quality by two independent authors and subsequently included for analysis. Data was extracted using standardized tools.

**Results:**

Narrative synthesis was undertaken to summarize and report the findings. This review demonstrates that the involvement of nurses in advanced practice in emergency and critical care improves the length of stay, time to consultation/treatment, mortality, patient satisfaction, and cost savings.

**Conclusions:**

Capitalizing on nurses in advanced practice to increase patients’ access to emergency and critical care is appealing. This review suggests that the implementation of advanced practice nursing roles in the emergency and critical care settings improves patient outcomes. The transformation of healthcare delivery through effective utilization of the workforce may alleviate the impending rise in demand for health services. Nevertheless, it is necessary to first prepare a receptive context to effect sustainable change.

**Electronic supplementary material:**

The online version of this article (10.1186/s12960-017-0237-9) contains supplementary material, which is available to authorized users.

## Background

While people of all ages receive emergency and critical care services across the world, the elderly population continues to exhaust a greater proportion of these services [1]. The complexity and acuity of care have heightened with greater prevalence of chronic illness and multimorbidity among older adults [2]. Correspondingly, the demand for emergency and critical care services has increased [1], alongside a concomitant increase in the forecasted workforce requirements for such services [3]. The Accreditation Council for Graduate Medical Education regulations in 2006 in the United States of America (USA) recommends a high-intensity model of care involving 24-h physician coverage [3, 4]. This implementation accentuates inadequacies of the healthcare workforce to provide emergent and critical care services. In the USA, it is predicted that, compared to healthcare system’s demands, there will be a 22% shortfall of critical care physicians by 2020 and a subsequent 35% shortfall by 2030 [1].

With the impending rise in demand for health services, an effective utilization of the workforce is paramount to ensure high-quality yet cost-effective health service delivery [5]. Across some countries, healthcare workers’ wages account for approximately 50% of the total healthcare expenditure [6]. Hence, cost containment strategies will inevitably involve the workforce [7]. Efforts are underway for measures to enhance productivity through increasing the capacity of the workforce.

One potential measure is a greater utilization of nurses in advance practice. The global annual growth of the nurse practitioner (NP) workforce has been estimated to be between three to nine times greater compared to physicians; therefore, of interest to health policymakers is the utilization of NPs and advanced practice nurses (APNs) [8, 9]. The nomenclature varies internationally. The “NP” title is used in Australia, Belgium, Canada, Sweden, the United Kingdom (UK), and the USA whereas the “APN” title is used in Switzerland, Singapore, and South Korea [10]. Nonetheless, NPs and APNs (NP/APNs) are registered nurses “who acquired the expert knowledge base, complex decision-making skills and clinical competencies for expanded practice” ([4], p. 26) and enter the workforce with a master’s degree [11].

This advanced practice role was first introduced in the 1960s as a solution to the lack of primary care physicians, to meet the primary care needs of the rural and underserved populations [12]. Primary care has first contact with patients and, subsequently, provides continuity of care within the healthcare system through the coordination of care according to patients’ needs [13]. Studies to evaluate the quality of primary care provided by NP/APNs have been shown to be comparable to that of physicians in terms of effectiveness and safety [14]. To fulfill primary care needs, NP/APNs in this setting are trained generalists who have a breadth of knowledge to render a wide scope of care.

Since the inception of advanced nursing practice in primary care, its role has extended to other healthcare settings such as the acute care. Acute care provides short-term restorative stabilization to patients in unstable chronic conditions and with complex acute and critical illnesses. Acute care encompasses emergency and critical care [15]. Emergency and primary care advanced nursing practice do share similarities in that they serve as first-contact access to healthcare, but the acuity of the patient manifestations delineates the two. Unlike in primary care NP/APNs, emergency NP/APNs are trained to manage patients with acute life- or limb-threatening conditions [15]. In the past decade, greater practice autonomy has been given to NP/APNs in emergency and critical care. This expanded practice allows nurses to assume some medical tasks typically performed by physicians, aiming at not only increasing the access to healthcare and service efficiency but also eventually mitigating the cost of health services.

The development of advanced nursing practice contributed to a service model aiming to respond flexibly to the ever-changing needs of patients [16]. Systematic reviews of studies on the effectiveness and safety of NP/APN-led primary care have reported positive effects of NP/APN service on clinical outcomes, patient satisfaction, and costs [14, 17] These reviews focused on the primary care setting, it may be inappropriate to extrapolate their findings to the emergency and critical care settings since the patient acuity and clinical needs differ among settings.

Nonetheless, reviews evaluating NP services in the emergency and critical care settings exist. However, they have three shortcomings, the first of which concerns their generalizability. Over the past decade, studies have evaluated whether the delegation of medical tasks to NP/APNs in the emergency and critical care settings was feasible and safe. A review of 31 studies on the impact of NPs and physician assistants in such settings reported that their practice was safe and, in some cases, the quality of care was higher than that of physicians [18]. However, only two of the studies were randomized controlled trials (RCTs) [19, 20] whereas the rest had small sample sizes and questionable study methodology; these limit the generalizability of the review. A more recent review [21] also reported that NPs do have a positive impact on the quality of care. Nonetheless, the reviews included both NPs and non-nursing healthcare providers, thereby introducing heterogeneity in the synthesis of evidence, making it difficult to assess the true effect of NPs in the intensive care settings [18, 21].

The second shortcoming centers on the inconclusiveness of the reviews. One review suggested although NP services in the emergency setting did reduce waiting time and provide care comparable to that of a midgrade physician, the cost of NP services was higher than that of resident physicians [22]. In contrast, another review concluded that the use of NPs reduced the cost of emergency and intensive care services. Further complicating the picture is a recent systematic review that reported an inadequacy of evidence to determine the cost-effectiveness of NP services in emergency departments (EDs) [23]. Consequently, the cost-effectiveness of advanced nursing practice in the emergency and critical care settings has remained inconclusive. Lastly, all existing reviews [18, 21–23] elucidating advanced nursing practice in the emergency and critical care settings included only studies published before January 2013, which may be dated.

Considering the existing literature, it is of interest to undertake an updated systematic review on the latest evidence to determine whether advanced practice nursing in emergency and critical care have an impact on the quality of care, clinical outcomes, patient satisfaction, and cost savings. If NP/APNs can indeed provide competent and safe care in these settings, greater access to emergency and critical care services will be available, thereby strengthening the workforce to fulfill the escalating healthcare demands.

Therefore, the main objective of this systematic review is to present, critically appraise, and synthesize the best available evidence on the impact of advanced nursing practice on patients’ length of stay, time to treatment or consult, mortality, patient satisfaction, and cost in emergency and critical care settings.

## Methodology

### Design

The Preferred Reporting Items for Systematic Reviews and Meta-Analyses (PRISMA) guidelines were adhered to in the conduct and reporting of this systematic review [24].

### Study selection

Published studies and studies which have yet to be published were searched using PubMed, CINAHL, The Cochrane Library, Scopus, Embase, Web of Science, ScienceDirect, Wiley Online Library, and ProQuest Dissertations and Theses Global databases from January 2006 up to September 2016. Only English studies were considered. The search strategy included the keywords, as shown in Table 1, in various combinations for a systematic database search. The search terms and search strategies for each database are included in Additional file 1. The reference lists of all identified studies were also screened. Corresponding authors were contacted for additional information where necessary.

**Table 1:** Summary of the themes and key words employed in the systematic review

Nurse	Physician-substitution	Setting	Outcome
Nurse practitioner* Nurse clinician* Non-physician Advance* practice nurs* Advance* nurs* pract*	Physician* Doctor* Medical practitioner* Interdisciplin* Case manage* Cooperative behav* Physician-Nurse	Intensive care unit Intensive care Critical care unit Critically ill* Subacute care High dependency care High dependency unit Emergency Acute care Acute disease Acute illness Trauma Post-operat*	Patient management Patient outcome Treatment Outcome Patient satisfaction Hospitalization Patient Readmission Mortality Hospital Cost* Clinical Competence Survival Time Factor* Staffing* Schedul* Workload Efficienc* Length of stay Wait* time Complication rate* Complication* Quality of care Cost* of care

*Denotes the use of a wildcard symbol to broaden the search to include variations on a distinctive word stem or root

### Study eligibility

This review included RCTs, quasi-experimental studies, prospective and retrospective cohort studies. Cross-sectional studies and studies without comparison groups were excluded.

The PICO (Population-Intervention-Comparison-Outcome) framework guided the selection process [25]. This review considered studies that included the following:

- Patients: at least 16 years of age, presenting in EDs, trauma centers, intensive care unit (ICU), or high dependency units, requiring emergency or critical care
- Nurses: registered nurses in advanced practice role, i.e., APNs or NPs
- Physicians: emergency physicians, intensivists, residents, medical officers, hospitalists, or house officers in the ED or ICU or high dependency units

Excluded from the review were studies that examined both adult and pediatric patients requiring emergency or critical care services. Excluded from the review were also studies that examined services provided by physician assistants. This review included studies with interventions which compared the outcomes of the APN-/NP-directed emergency or critical care services with those of the physician-directed care. This review also included studies with interventions which compared the physician-only model of care with APN-physician or NP-physician collaborative model of care.

Studies that had the following outcome measures were included:

- Patients’ length of stay in the emergency or critical care setting
- Patient mortality
- Time to consultation or treatment
- Patients’ satisfaction
- Cost of care

**Fig. 1:**
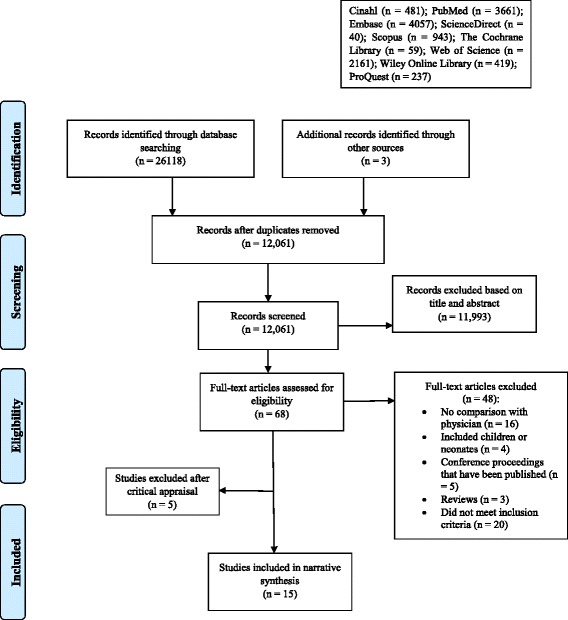
Systematic review search flow diagram

The selection of studies was done independently by two of the authors (BW and JL) based on the eligibility criteria. Disagreement during selection was resolved by discussion with a third-party arbiter (WT). The selection process is illustrated in the flow diagram in Fig. 1.

### Data extraction

Data was extracted by one author (BW) and crosschecked by another (JL) for accuracy. Resolution of disagreement was done by discussion with a third-party arbiter (WT). The Joanna Briggs Institute’s (JBI) “Data Extraction Form for Experimental/Observational Studies” [26] was adapted to tabulate the characteristics and findings of the studies.

### Quality assessment

Two authors (BW and JL) performed the methodological quality assessment independently, based on the “JBI Critical Appraisal Checklist for Randomized Controlled Trials,” and “JBI Critical Appraisal Checklist for Cohort Studies” [21]. The RCTs were assessed for their randomization methods, treatment allocation, concealment of treatment groups, and homogeneity of the participants’ baseline demographics upon entry of the study. In addition, all studies were appraised for their control of confounding factors, reliability of outcome measures, and suitability of statistical analyses. For this review, a low methodological quality refers to a score assigned to a study of less than 40%, a medium quality refers to one between 40 and 70%, and a high quality refers to one greater than 70%. The findings of any systematic review are only as reliable as the primary data source, upon which the review is based [27]. Hence, studies rated to have low methodological quality (see Additional file 2) were excluded to avoid potentially erroneous conclusions based on the synthesis of poorly conducted studies.

### Synthesis

Given the heterogeneity of the interventions and findings in the studies, no meta-analysis was performed. Instead, a narrative synthesis of the studies was done: the analysis was conveyed in prose, alongside tables to outline and explain the results.

## Results

### Study characteristics

This review included 15 studies with 23 681 participants across five countries including Australia [28–30], Canada [31, 32], New Zealand [33], UK [34], and USA [35–41], where the nomenclature for nurses in advanced practice was “NP.” A total of 14 studies [28–41] were published while one was an unpublished manuscript (Roche T, Gardner GE, Jack L: The effectiveness of emergency nurse practitioner service in the management of patients presenting to rural hospitals with chest pain: a multisite prospective longitudinal nested cohort study. In preparation.) at the point of the search. The previously unpublished manuscript was subsequently published in 2017 [42]. All included studies were conducted between 2006 and 2016. As regards the setting, six studies [28–30, 32, 33] focused on the EDs, six [31, 34, 35, 37, 38, 41] on the ICU, two [36, 40] on the trauma centers, and one on the stroke center [39]. The sample sizes ranged from 103 [31] to 9066 [38]. The characteristics of the studies are detailed in Table 2.

**Table 2:** Characteristics of study

Reference, country	Study quality	Study objective	Setting	Study design	Participant	Comparison groups	Intervention	Outcomes measured
Colligan et al. (2011), New Zealand [33]	High	To determine if emergency NPs (ENPs) were equivalent to emergency medicine (EM) registrars in managing minor injuries	ED of a tertiary hospital	Prospective cohort study	Patients > 15 years presenting with trauma (*n* = 420)	Intervention (*n* = 305): ENP. Median age 30; 70% male; 62% Caucasian; 81% triage 4; 35% procedures performed. Comparator (*n* = 115): EM registrars. Median age 41; 59% male; 66% Caucasian; 72% triage 4; 32% procedures performed.	ENP managed minor injuries. ENP administered anesthetic and rendered treatment procedure as required independently.	ED length of stay (LOS)
David et al. (2015), USA [35]	Medium	To determine if the addition of a cardiac acute care NP (ACNP) to care teams could improve utilization outcomes	Cardiovascular ICU (CCU) of a large urban and academic medical center	Retrospective cohort study	Patients admitted directly to the CCU with the primary diagnosis of either ST or non-ST segment elevation myocardial infarction (non/STEMI) or heart failure (HF) (*n* = 185)	Intervention (*n* = 109): Cardiac ACNP in collaboration with CCU physician house staff team. Mean age 69.2; 62.4% male; 28.4% HF; 71.6% non/STEMI. Comparator (*n* = 76): CCU physician house staff team. Mean age 70.6; 65.8% male; 26.3% HF; 73.7% non/STEMI.	Cardiac ACNP and physician worked together within a multidisciplinary team. Responsibilities of ACNP include routine medical care, discharge planning, care coordination, patient education on disease process and self-care, and post-discharge telephone follow-ups.	30-day return to ED; 30-day readmission rate; LOS; time of discharge
Dinh et al. (2012), Australia [30]	Medium	To compare the quality of care provided by an ENP and emergency doctors	Fast-track unit within the ED of a suburban hospital	RCT	Patients between age 16 and 70 years presenting to the ED with Australasian Triage Scale (ATS) category 4 or 5, who had normal vital signs and mental state, without complex medical or surgical comorbidities, and did not require multiple diagnostic tests or specialty consultations (*n* = 233)	Intervention (*n* = 133): ENP. Median age 37; 60% male; 73% musculoskeletal presenting problem. Comparator (*n* = 103): ED doctors ranged from resident medical officers, emergency registrars, career medical officers, and emergency physicians. Median age 33; 64% male; 71% musculoskeletal presenting problem.	ENP worked independently, assessed and managed patients within the fast-track unit, and consulted senior medical staff when required.	Patient satisfaction scores; follow-up health status at 2-week follow-up; adverse events (readmission to ED within 14 days or missed fractures); waiting time to be seen
Goldie et al. (2012), Canada [31]	Medium	To compare the effectiveness of ACNP-led care to hospitalist-led (physicians trained in general medicine) care in a post-cardiac surgery patients	Post-operative cardiac surgery unit in a tertiary hospital	RCT	Patients ≥ 18 years who had been scheduled for either urgent or elective coronary artery bypass graft (CABG) and/or valvular surgery (*n* = 103)*.*	Intervention (*n* = 22): ACNP-led post-operative care, guided by previously established clinical pathway. Mean age 67; 86% male; 85% urgent procedure; 71% CABG. Comparator (*n* = 81): Hospitalist-led post-operative care, guided by previously established clinical pathway. Mean age 65; 81% male; 43% urgent procedure; 62% CABG.	The ACNP functioned solely as a clinician, performs focused physical assessments and comprehensive health history-taking, and reviewed the patients’ medications and diagnostic tests to develop care plans for the patients to augment established clinical pathway. Upon discharge, the ACNP communicated with the family physician of patients whom she anticipated complications post-discharge to discuss plan of care for the patient.	LOS; hospital readmission within 60 days; post-operation complications; attendance at cardiology or cardiac rehabilitation appointments; overall patient satisfaction; overall team satisfaction
Hiza et al. (2015), USA [36]	Medium	To analyze the effect of an orthopedic trauma NP on LOS and cost	Level I trauma center	Retrospective cohort study	Patients who were treated operatively and non-operatively or who were transferred from other services to the orthopedic trauma team and who were then discharged from the orthopedic trauma team (*n* = 1 584)	Intervention (*n* = 871): NP as an additional member of the orthopedic trauma team. 80.25% <60 years; 64.41% ED admission. Comparator (*n* = 713): Orthopedic trauma team without NP. 85.27% <60 years; 76.6% ED admission.	A single full-time NP added to the orthopedic trauma team. The NP assisted the orthopedic intern in daily floor work such as arranging social service needs, discharge planning, and paperwork. The NP acted as a liaison for the orthopedic trauma team in daily multidisciplinary meetings between other physicians, allied health professionals, nurse managers, and social workers.	LOS; cost
Hoffman et al. (2006), USA [37]	Medium	To compare the outcomes of patients when medical management was provided by an attending physician in collaboration with a unit-based ACNP or an attending physician and critical care/pulmonary care fellows who rotated coverage	Subacute medical ICU (MICU) of a university medical center	Prospective cohort study	Patients admitted to the subacute MICU who required prolonged mechanical ventilation (≥ 7 days) with tracheostomy (*n* = 192)	Intervention (*n* = 98): An attending physician in collaboration with a unit-based ACNP. Mean age 61.9; 51% male; 85.6% white; 56.1% acute pulmonary diagnosis. Comparator (*n* = 94): An attending physician and critical care/pulmonary care fellows who rotated coverage. Mean age 61.2; 53.2% male; 87.1% white; 48.9% acute pulmonary diagnosis	The ACNP was responsible for assessment, diagnosis, and documentation of patient care, including weaning and extubation. The ACNP was responsible for the admission of patients and discharge decisions. During the rounds, the attending physician would review and revised the plan of care.	ICU LOS; days on mechanical ventilation; readmissions to MICU; ICU mortality
Jennings et al. (2008), Australia [28]	Medium	To assess the impact of the implementation of ENP candidate (ENPC) on waiting times and LOS for patients presenting to the ED	Emergency and trauma center	Retrospective cohort study	Adult patients in ATS categories 3 to 5 (*n* = 3 156)	Intervention (*n* = 572): ENPC completed care of patient. 6.1% ATS 3; 63.7% ATS 4; 30.2% ATS 5. Comparator (*n* = 2 584): Medical officer completed care of patient with assistance from nurses. 19.5% ATS 3; 58.4% ATS 4; 22.1% ATS 5.	ENPC are nurses who are practicing within the role and seeking accreditation as NPs. The ENPC completed the care for each presenting patient from initial assessment, intervention, prescribing, diagnosis, treatment, and disposition within a collaborative ED team using Clinical Practice Guidelines for each presentation.	LOS; time to be seen
Jennings et al. (2015), Australia [29]	High	To compare the effectiveness of NP service with standard medical care in the ED	ED of a major referral hospital	Pragmatic RCT	Adult patients presenting with verbal numeric pain scale score > 1 and in ATS categories 2 to 5 (*n* = 258)	Intervention (*n* = 130): NPs managed patient care with assistance if necessary from a registered nurse. Mean age 30; 53% male; 66% ATS 4. Comparator (*n* = 128): Medical officers managed patient care with assistance from a registered nurse. Mean age 33; 61% male; 63% ATS 4.	The ENP manages the care of the patient. After the initial assessment, the ENP initiated the management of the patient and completed the episode of care. Analgesics were prescribed by NPs when required.	Proportion of patients who received analgesia within 30 min; time to analgesia from ED arrival; changes in pain score; documentation of pain scores
Landsperger et al. (2016), USA [38]	High	To evaluate the safety of the continuous in-house ACNP care as compared to in-house resident care	MICU of a university hospital	Prospective cohort study	Adult patients admitted to a MICU team (*n* = 9 066)	Intervention (*n* = 2366): Team led by ACNP, supervised by critical care fellows and attending physicians. Mean age 55.9. 51% male; 78% Caucasian; 53% ED admission; 28% mechanical ventilation; 27% vasopressors. Comparator (*n* = 6 700): Team led by 1st year resident and 1 upper level resident, supervised by critical care fellows and attending physicians. Mean age 56.7; 52% male; 76% Caucasian; 52% ED admission; 33% mechanical ventilation; 36% vasopressors.	The ACNP was responsible for the evaluation and management of patients. Responsibilities included conducting admissions, transfers, discharges, obtaining and interpreting diagnostic tests, and performing critical care procedures with supervision of critical care fellows and attending physicians.	90-day survival; ICU LOS; hospital LOS; ICU mortality; hospital mortality; longer term mortality
Moran et al. (2016), USA [39]	Medium	To evaluate if the introduction of 24/7, on-site coverage with a neurocritical ACNP as first responders for acute “stroke code” would shorten time to treatment and improve compliance with acute stroke time targets	Stroke center of a tertiary hospital	Retrospective cohort study	Adult patients with the principal diagnosis of acute ischemic stroke (*n* = 168)	Intervention (*n* = 122): On-call neurovascular physician and 24/7 ACNP first responder coverage for the hospital stroke code team. Median age 73; 49% male; 48% Asian; 77% hypertension. Comparator (*n* = 44): On-call vascular neurologist or neurointensivist had a 30-min window for arrival to the bedside after the stroke code team was activated. Median age 68; 54% male; 48% Asian; 77% hypertension.	The ACNP took initial history, obtained the National Institutes of Health Stroke Scale (NIHSS) score, obtain and review imaging, review the indications and contraindications for tissue plasminogen activator (tPA), and discussed tPAeligibility with the on-call vascular neurologist by telephone. For patients who were ineligible for tPA, the ACNP documented the clinical encounter. For patients who were eligible for tPA, the on-call vascular neurologist directly evaluated the patient and made the final decision regarding tPA administration.	Onset-to-needle time; imaging-to-needle time; door-to-needle time; hospital mortality
Morris et al. (2012), USA [40]	High	To determine if there were differences between the care provided by unit-base NP (UBNP) and residents	Level 1 trauma center	Retrospective cohort study	Adult patients requiring trauma service (*n* = 3 859)	Intervention (*n* = 2 759): UBNP care of trauma patients led by trauma attending physicians. Mean age 42.4; 72% male; 52% African American. Comparator (*n* = 1 100): Resident care of trauma patients led by trauma attending physicians. Mean age 42.6; 70% male; 54% African American.	A group of NPs provided direct daily care, supervised by the trauma attending physician. Resident involvement with the patients admitted to the UBNP floor is limited to invasive procedures and overnight cross-coverage.	ICU admission; LOS; complications; readmissions
Roche et al. (2017), Australia [42]	Medium	To examine the safety and quality of ENP service in the provision of care and the effectiveness of ENP service for adults with chest pain	EDs of 3 rural hospitals	Prospective cohort study	Patients ≥ 18 years presenting with chest pain that was not a result of an acute injury (*n* = 61)	Intervention (*n* = 23): ENP model. Mean age 59.9; 30% male. Comparator (*n* = 38): Standard care model (care delivered and coordinated by medical officer). Mean age 61.7; 50% male.	The ENP managed the patient presenting with undifferentiated chest pain. The ENP delivered and coordinated care in diagnosis, investigation, therapeutic treatment, and referral.	Adherence to guidelines; diagnostic accuracy of ECG interpretation; waiting times; LOS; LWOT; diagnostic accuracy as measured by unplanned representation rates; patient satisfaction; quality-of-life; functional status
Scherzer et al. (2016), USA [41]	Medium	To compare usage patterns and outcomes of a NP-staffed MICU and a resident-staffed physician MICU	MICU of a large urban university hospital	Retrospective cohort study	Patients admitted to the adult MICU (*n* = 1 157)	Intervention (*n* = 221): NP-staffed MICU. Mean age 62.3; 53.8% male; 64.3% White; 39.4% respiratory failure. Comparator (*n* = 936): Resident-staffed MICU. Mean age 59.2; 55.8% male; 56.1% White; 32.8% respiratory failure.	Daytime staffing consisted for 2 internal medicine residents and two NPs, supervised by an attending critical care physician. Nighttime coverage consisted of 1 NP with 1 critical care fellow.	MICU mortality; hospital mortality; MICU readmission; MICU LOS; hospital LOS; post-MICU discharge LOS; charges observed
Skinner et al. (2013), UK [34]	Medium	To assess the feasibility and safety of NPs providing first-line care on an ICU with all doctors becoming non-resident at night	Cardiac ICU of a tertiary hospital	Retrospective cohort study	Patients admitted to an adult cardiac ICU (*n* = 1 380)	Intervention (*n* = 678): NP providing first-line care. Comparator (*n* = 702): Junior resident doctors providing first-line care.	Model of care included NPs in the team and resident NP providing first-line care after evening rounds. Non-resident doctors remain within 15 min of the hospital.	ICU mortality; annual staffing cost
Steiner et al. (2009), Canada [32]	Medium	To determine if the addition of a broad-scope NP would improve wait times, ED LOS and left-without-treatment (LWOT) rates	Urban community ED	Prospective cohort study	Patients requiring ED services (*n* = 3 238)	Intervention (*n* = 1 924): NP collaborative visits or NP autonomous visits. Comparator (n = 1 314): Emergency physician (EP) visits.	The NP collaborative model was like that of residents, with the EP retaining the ultimate decision-making authority. The NP also provided health promotion and counseling. EP delegated specific discretionary tasks such as direct patient care, discharge planning and follow-up arrangements to an NP. In the NP autonomous scope of practice, it was limited to patients in categories 4 and 5 of the Canadian ED Triage and Acuity Scale (CTAS).	Wait times; ED LOS; LWOT

### Methodological quality

The assessment details of each study’s methodological quality are presented in Table 3. In this review, only three studies were RCTs [29–31] whereas 12 were cohort studies [28, 32–42]. The included studies had low to medium risk of bias.

**Table 3:** Summary of methodological quality of included studies

Methodological quality of the randomized controlled trials											
	Was true randomization used for assignment of participants to treatment groups?	Were treatment groups similar at the baseline?	Were outcomes assessors blind to treatment assignment?	Were treatments groups treated identically other than the intervention of interest?	Was follow-up complete, and if not, were strategies to address incomplete follow-up utilized?	Were participants analyzed in the groups to which they were randomized?	Were outcomes measured in the same way for treatment groups?	Were outcomes measured in a reliable way?	Was appropriate statistical analysis used?	Was the trial design appropriate, and any deviations from the standard RCT design accounted for in the conduct and analysis of the trial?	Quality
Dinh [30]	+	+	−	+	−	+	+	?	+	+	Medium
Goldie [31]	?	−	+	+	−	+	+	+	+	+	Medium
Jennings [29]	+	+	+	+	+	+	+	+	+	+	High
Methodological quality of the cohort studies											
	Were the groups similar and recruited from the same population?		Were the exposures measured similarly to assign people to both exposed and unexposed groups?	Were confounding factors identified?	Were strategies to deal with confounding factors stated?	Were the outcomes measured in a valid and reliable way?	Was follow-up complete, and if not, were the reasons to loss to follow-up described and explored?		Were strategies to address incomplete follow-up utilized?	Was appropriate statistical analysis used?	Quality
Colligan [33]	−		+	+	+	+	+		N.A.	+	High
David [35]	+		+	+	−	+	−		−	+	Medium
Hiza [36]	?		+	+	+	+	?		?	+	Medium
Hoffman [37]	−		+	+	−	+	?		?	+	Medium
Jennings [28]	−		+	+	+	+	?		?	+	Medium
Landsperger [38]	−		+	+	+	+	+		N.A.	+	High
Moran [39]	+		+	+	−	+	?		?	+	Medium
Morris [40]	+		+	+	−	+	+		N.A.	+	High
Roche [42]	+		+	+	−	+	?		?	+	Medium
Scherzer [41]	−		+	+	−	+	?		?	+	Medium
Skinner [34]	+		+	−	−	+	?		?	+	Medium
Steiner [32]	?		+	+	+	+	−		−	+	Medium

+ yes; − no; ? unsure

In two of the three RCTs, true randomization was used to assign patients to study groups by using computer-generated sequence, thus incurring only low risk of selection bias. In the other RCT, a triage coordinator was present to randomly assign the patients at a planned ratio to either NP-directed care or physician-directed care. Two of three RCTs, took measures to blind the outcome assessors to treatment assignment, minimizing detection bias. Out of the 15 studies, 14 measured their outcomes in a reliable and valid manner using pre-decided criteria, minimizing reporting bias. The presence of confounding factors was acknowledged in 11 of the 12 cohort studies but only five of them described strategies to deal with it. All the included studies fared poorly in reducing attrition bias. Only three of the 15 studies had complete follow-up or strategies to address incomplete follow-up. Appropriate statistical analyses were chosen in all included studies.

### Findings

The study results and statistical conclusions are summarized in Table 4. The details of the individual studies can be found in Table 5. The findings were categorized according to the studies’ setting. Studies conducted in emergency and critical care settings measured outcomes such as length of stay, waiting, and patient satisfaction. Outcomes such as mortality and cost were measured only in the critical care setting.

**Table 4:** Summary of study results and statistical conclusions by outcome

Study	Setting	Length of stay	Waiting time		Mortality	Patient satisfaction	Cost
			Time to consultation	Time to treatment			
NP-directed care (NP only)							
Colligan [33]	ED	↓	↓				
Dinh [30]	ED		↔			↑	
Goldie [31]	Post-cardiac surgery unit	↔				↔	
Jennings [28]	ED	↓	↔				
Jennings [29]	ED			↓			
Landsperger [38]	ICU	↓(ICU) ↓(Hospital)			↔		
Moran [39]	Stroke center			↓	↔		
Morris [40]	Trauma center	↔					
Roche [42]	ED	↔	↔			↔	
Collaborative care (NP + Physician)							
David [35]	ICU	↔					
Hiza [36]	Trauma center	↔					↓
Hoffman [37]	ICU	↔			↔		
Scherzer [41]	ICU	↑(ICU) ↔(Hospital)			↔		↔
Skinner [34]	ICU				↔		↓
Steiner [32]	ED	↔	↔				

↑significant increase; ↔ no significant difference; ↓significant decrease

**Table 5:** Findings of studies

Outcome measured	Results	Interpretation
Length of stay (LOS)—Emergency setting		
Colligan et al. (2011) [33]	For patients who underwent procedures for their minor injuries, significant difference between study groups in the median LOS was present, 92 min (IQR 62–132) in NP group versus 135 min (96–200) in Registrars group (Mann-Whitney *U* test *P* < 0.0001). For patients who did not undergo any procedures, significant difference between study groups in the median LOS was also present, 119 min (IQR 68–154) in NP group versus 135 min (118–214) in Registrars group (Mann-Whitney *U* test *P* < 0.0002).	• A New Zealand study conducted at a single site. • Registrars took a longer time to see these minor injuries patients as the patients were of higher acuity with comorbidities while the ENP reviewed the straightforward minor injury cases. • NPs tend to complete patient care on their own while Registrars would delegate discharge or administrative tasks to clerical staff. • The time recorded electronically might not have been precise in reflecting the patient’s transit times. It was possible NPs logged onto the system to review patients faster than Registrars which might have account for the reduced LOS for NP-treated patients.
Jennings et al. (2008) [28]	Significant difference between study groups in the median ED LOS, 94 min (IQR 53.5–163.5) in the ENP candidate group versus 170 min (IQR 100–274) in the medical officers group (Wilcoxon *P* < 0.001).	• An Australian study conducted at a single site. • Patients in the ENP candidate group were from the Fast Track unit where patients of lower acuity were seen. Patients in the medical officers group were not only from the Fast Track unit. The medical officers might have reviewed more complex cases and hence, required more time.
Roche et al. (2017) [42]	No significant difference between study groups in median LOS, 97.0 min (IQR 91) in NP group versus 101.5 min (IQR 54) in medical officer group (Mann-Whitney *U* test *P* = 0.8).	• An Australian study conducted at three rural EDs. • Small sample size, underpowered study. • No significant differences between groups in baseline characteristics or acuity, NP service was comparable to that of senior medical officers.
Steiner et al. (2009) [32]	No significant difference between study groups in median ED LOS, 125 min (IQR 78–192) in NP group versus 123 min (IQR 76–184) in physician group (Wilcoxon *P* = 0.13).	• A Canadian study conducted at a single site. • The emergency physician group had patients of higher acuity than NP collaborative group yet there was no difference in LOS between groups, possibly implying it was more efficient to do without collaboration with NPs. • However, the demand for physicians to review lower acuity patients might have reduced with the collaborative NP group, allowing physicians to spend more time with higher acuity patients.
Length of stay (LOS)—Critical Care setting		
David et al. (2015) [35]	No significant difference found between study groups in the mean LOS in the inpatient telemetry cardiology unit and ICU, 129.1 ± 96.7 h in NP collaborative group versus 119.1 ± 69.7 h in physician-only group (*P* = 0.469).	• A USA study conducted at a single site. • Advocates for the NP collaborative model of care as it provides the unit staff with a consistent point of contact for the multidisciplinary team. • The NP collaborative model of care allows for NPs to develop expertise for managing a specific group of patients.
Goldie et al. (2012) [31]	No significant difference found between study groups in the mean hospital LOS, 9 ± 6 days in NP group versus 9 ± 14 days in hospitalist group (*t* test, *P* = 0.87).	• A Canadian RCT conducted at a single site. • Total sample size varied during the statistical analysis as there were varying amounts of missing data. • A much higher proportion of male participants recruited (86% in NP group and 81% in hospitalist group) raised queries about system level factors that might have favored male participants and the general willingness of female patients to participate in research. • The patient acuity in NP group was higher than that in hospitalist group and yet the groups did not differ in their clinical outcomes.
Hiza et al. (2015) [36]	No significant difference found between study groups in mean LOS, 4.91 ± 4.53 days in the NP collaborative group versus 6.02 ± 6.74 days in the physician group (Wilcoxon *P* = 0.1441).	• A USA study conducted at single site. • After subgroup analysis, significant differences in LOS were found between study groups in patients transferred from another service (Wilcoxon *P* < 0.0001), patients discharged to rehabilitation facility (Wilcoxon *P* = 0.0024), patients older than 60 years (Wilcoxon *P* = 0.0369), or patients discharged on intravenous antibiotics/wound therapy (Wilcoxon *P* = 0.0171). A significantly lower mean LOS was found in the NP collaborative group. • In this subgroup of patients, greater communication with multidisciplinary teams, discharge planning, follow-up care coordination and administrative work were required. This demonstrated the value of adopting the NP collaborative model of care.
Hoffman et al. (2006) [37]	No significant difference between study groups in the mean ICU LOS, 14.6 ± 9.7 days in NP collaborative group versus 15 ± 11.4 days in non-NP group (*P* = 0.753).	• A USA study conducted at a single site. • The comparable ICU LOS between NP collaborative model of care and the model of care without NP might be due to the greater continuity of care rendered by the NP as compared to the rotating coverage of the fellows in the non-NP model of care. • It might also be contributed by the attending physician’s ability to provide expert supervision and direct care of the patients, despite the difference in the composition of the team. • It could also be because the NP was highly experienced and was familiar with the environment and the patient care demands.
Landsperger et al. (2016) [38]	Significant difference between study groups in median ICU LOS, 3.4 ± 3.5 days in NP group versus 3.7 ± 3.9 days in Resident group (Wilcoxon *P* < 0.001). Similar odds of a longer ICU stay between groups (odds ratio 1.01, 95% CI 0.93–1.1, *P* = 0.81) Significant difference between study groups in median hospital LOS, 7.9 ± 11.2 days in NP group versus 9.1 ± 11.2 days in Resident group (Wilcoxon *P* < 0.001). NP group had lower odds of a longer ICU stay compared to Resident group (odds ratio 0.87, 95% CI 0.80–095 *P* = 0.001).	• An USA study conducted at a single site. • Large prospective cohort study (*n* = 9066). • Patients in NP group were solely managed by NPs and the supervising attending physicians and fellows. There was no cross-contamination, the Residents did not interfere with the management of patients in the NP group. • Even though LOS findings between study group favor the NP group, the lack of clear definition of the role of the acute care NP hinders direct comparison of clinical outcomes with the residents. • Hospital LOS for NP group was shorter than Resident group as more patients were being discharged straight from the ICU in NP group. It could have been due the differences in patient’s diagnosis, social or financial situations, or provider practice paradigm. • Shorter hospital LOS in NP group did not come at the expense of longer ICU LOS, increased ICU readmissions or post-discharge mortality. • A higher patient to provider ratio was observed in NP group but the authors were judicious in inferring that NP-led model of care had greater efficiency given the differences in the patients’ characteristics between study groups.
Morris et al. (2012) [40]	No significant difference between study groups in mean LOS, 6.5 ± 8.8 days for NP group versus 7 ± 10.8 days for Resident group (*t* test *P* = 0.17).	• A USA study conducted at a single site. • Although the results are not statistically significant, they were clinically important. The difference of 0.5 days multiplied by the number of patients in NP group (2759) accumulates to a total difference of greater than 1300 patient days. • A greater proportion of Resident group discharged to other health facilities which was delayed by bed availability. This could be a possible reason for the longer hospital LOS for patients in Resident group. • Daily multidisciplinary rounds were scheduled in NP group but not in Resident group which could have improved the coordination of patient care, contributing to shorter LOS.
Scherzer et al. (2016) [41]	Significant difference between study groups in mean MICU LOS, 7.9 ± 7.5 days in NP group versus 5.6 ± 6.5 days in Resident group (Wilcoxon *P* < 0.0001). No significant difference between study groups in mean hospital LOS, 18.0 ± 16.8 days in NP group versus 15.9 ± 19.9 days in Resident group (Wilcoxon *P* = 0.435). No significant difference between study groups in mean post-MICU discharge LOS, 6.4 ± 8.7 days in NP group versus 8.4 ± 15.6 days in Resident group (Wilcoxon *P* = 0.102).	• A USA study conducted at a single site. • Presence of differing clinical practice between NP and Residents could have contributed to the difference in MICU LOS. • Patients in NP group were older, more chronically and critically ill than patients in Resident group and so were more likely to require longer MICU care. • Patients in NP group had higher likelihood of being discharged to a post-acute care setting compared to patients in Resident group. The availability of the discharge facility could have attributed to MICU LOS.
Waiting time (Time to consultation/Time to treatment) – Emergency setting		
Colligan et al. (2011) [33]	Significant difference between study groups in median time to consultation, 14 min (IQR 5–27) in NP group versus 50 min (IQR 21–78) in Registrars group (Mann-Whitney *U P* < 0.0001).	• A New Zealand study conducted at a single site. • EM Registrars might have taken a longer time between each patient because they were of higher acuity and complexity compared to patients in NP group. • The time recorded electronically might not have been precise in reflecting the patient’s transit times. It was possible NPs logged onto the system to review patients faster than Registrars which might have account for the lesser wait times for NP-treated patients.
Dinh et al. (2012) [30]	No significant difference between study groups in median waiting time to be seen, 50 min (IQR 33–77) in NP group versus 57 min (IQR 31–110) in doctor group (*P* = 0.06).	• An Australian study conducted at a single site. • Lost to follow-up rates was high. The waiting time of patients who left before being seen was not captured. • Patients in both study groups had similar baseline characteristics. • Patients seen by NP and doctors had comparable waiting time to consultation.
Jennings et al. (2008) [28]	No significant difference between study groups in median time to consultation, 12 min (IQR 5.5–2.8) in the ENP candidate group versus 31 min (IQR 11.5–76) in medical officer group (Wilcoxon *P* < 0.001).	• An Australian study conducted at a single site. • Patients in the ENP candidate group were from the Fast Track unit where patients of lower acuity were seen. Patients in the medical officers group were not only from the Fast Track unit. The medical officers might have reviewed more complex cases and hence, required more time.
Jennings et al. (2015) [29]	Significant difference between study groups in the proportion of patients receiving analgesia within 30 min of ED arrival, 15.4% in NP group versus 1.6% in medical officer group (Chi-square test *P* < 0.001).	• An Australian study conducted at a single site. • NP group performed better at complying with the recommended Australian national targets for administering timely analgesia. • NP provided a hybrid model of care, assimilating nursing, and medical tasks. The NP could perform patient assessment, order and administer the analgesia which reduced the time to treatment.
Roche et al. (2017) [42]	No significant difference between study groups in median waiting time, 8 min (IQR 23) in NP group versus 7.5 min (IQR 20) in medical officer group (Mann-Whitney *U* test *P* = 0.4).	• An Australian study conducted at a single site. • Small sample size, underpowered study. • No significant differences between groups in baseline characteristics or acuity, NP service was comparable to that of senior medical officers.
Steiner et al. (2009) [32]	No significant difference between study groups in median time to consultation, 61 min (IQR 34–99) in NP group versus 65 min (IQR 35–105) in physician group (Wilcoxon *P* = 0.62).	• A Canadian study conducted at a single site. • The emergency physician group had patients of higher acuity than NP collaborative group yet there was no difference in waiting time between groups, possibly implying it was more efficient to do without collaboration with NPs.
Waiting time (time to consultation/time to treatment)—Critical Care setting		
Moran et al. (2016) [39, 40]	Significant difference between study groups in median door-to-needle time for acute ischemic stroke, 45 min (IQR 35–58) in NP group versus 53 min (IQR 45–73) in non-NP group (Mann-Whitney *U P* = 0.001).	• A USA study conducted at a single site. • Stroke code care pathway remained the same during the intervention period. • The reduced time interval between diagnostic imaging and the administration of treatment contributed to the reduction in door-to-needle time. • NP group was reviewed earlier upon stroke code activation as the NP service was 24/7. Necessary assessments commenced earlier.
Mortality—Critical Care setting		
Hoffman et al. (2006) [37]	No significant difference between study groups in ICU mortality, 2% in NP collaborative group versus 2% in non-NP group without treatment limitation (Fisher’s exact test *P* = 1.0).	• A USA study conducted at a single site. • The comparable ICU mortality between NP collaborative model of care and the model of care without NP might be due to the greater continuity of care rendered by the NP as compared to the rotating coverage of the fellows in the non-NP model of care. • It might also be contributed by the attending physician’s ability to provide expert supervision and direct care of the patients, despite the difference in the composition of the team. • It could also be because the NP was highly experienced and was familiar with the environment and the patient care demands.
Landsperger et al. (2016) [38, 65]	No significant difference between study groups in ICU mortality (adjusted odds ratio 0.77, 95% CI 0.63–.94, *P* = 0.1). No significant difference between study groups in hospital mortality (adjusted odds ratio 0.87, 95% CI 0.73–1.03, *P* = 0.11) No significant difference between study groups in 90-day mortality (adjusted odds ratio 0.94, 95% CI 0.83–1.07, *P* = 0.36). No significant difference between study groups in longer term mortality (adjusted odds ratio 1.03, 95% CI 0.92–1.1 *P* = 0.65).	• An USA study conducted at a single site. • Large prospective cohort study (*n* = 9066). • Cross-contamination was minimized, the Residents did not interfere with the management of patients in the NP group. Patients in NP group were solely managed by NPs and the supervising attending physicians and fellows. • The 90 days and beyond information on the patient’s outcome strongly suggests that the quality of NP services in the ICU setting is high.
Moran et al. (2016) [39]	No significant differences between study groups in hospital mortality, 12% in NP group versus 18% in non-NP group (chi-square test, *P* = 0.33).	• A USA study conducted at a single site. • Stroke code care pathway remained the same during the intervention period. • The involvement of the NP in the stroke code team did not change the overall tPA treatment rate of acute ischemic stroke patients because the final decision to treat lies with the stroke physician.
Scherzer et al. (2016) [41]	No significant difference between study groups in MICU, 14.5% in NP group versus 13.1% in Resident group (adjusted odds ratio 0.8, *P* = 0.441). No significant difference between study groups in hospital mortality, 24.4% in NP group versus 24.8% in Resident group (adjusted odds ratio 0.7, *P* = 0.072).	• A USA study conducted at a single site. • Patients in NP group were older, more chronically and critically ill than patients in Resident group yet the MICU and hospital mortality in both groups were comparable. • Administrative data was used to calculate the risk of mortality. Furthermore, only a subset of the patients had their ICU mortality score calculated. An exhaustive comparison to national data was not done. Outcomes were only compared within the single institution.
Skinner et al. (2013) [34]	No significant difference between study groups in ICU mortality, 2.8% in NP group versus 2.2% in junior resident group (chi-square test, *P* = 0.43).	• A UK study conducted at a single site. • The new model of care with NP providing first-line care was not inferior to that of usual model of care. • The junior surgeons had more training time in the operating theaters.
Patient Satisfaction – Emergency setting		
Dinh et al. (2012) [30]	Significant difference between study groups in overall rating categories. A higher proportion (68%) of patients in the NP group rated their care as excellent compared to the doctor group (50%) (Fisher exact test, *P* = 0.02). Significant difference between study groups in total patient satisfaction score, median score 23 (IQR 20–24) in NP group versus median score 21 (IQR 16–24) in doctor group (Students t test, *P* = 0.002).	• An Australian study conducted in a single site. • Loss to follow-up rates were high. The satisfaction level of these patients was not captured.
Roche et al. (2017) [42]	No significant difference between study groups in patient satisfaction of care at the occasion-of-service (Fisher’s exact test, *P* = 0.96). No significant difference between study groups in patient satisfaction of care at follow-up (Fisher’s exact tests, *P* = 0.98).	• An Australian study conducted at a single site. • Small sample size, underpowered study. • Evidence to show that majority of the patients were highly satisfied (88.5%) with NP services in the ED and was sustained over time (30 days).
Patient satisfaction—Critical Care setting		
Goldie et al. (2012) [31]	No significant difference between study groups in mean overall patient satisfaction score, 103 ± 11 in NP group versus 97 ± 14 in hospitalist group (independent *t* test, *P* = 0.10).	• A Canadian RCT conducted at a single site. • Although there was no significant difference between groups in overall patient satisfaction, patients rated NP services significantly higher on several patient satisfaction items. • The NPs were rated to perform better at teaching, answering questions, listening and pain management. • These are the forte of NPs, consistent with the NP goals and education, which are grounded in nursing. • The overall patient satisfaction score of NP group was higher than in the hospitalist group though not statistically significant, it was plausible Type II error happened, and a larger sample size would have generated statistical differences in the overall score.
Cost—Critical Care setting		
Hiza et al. (2015) [36]	Averagely, US$ 2 000 is incurred per day for hospitalization. For the subgroup of patients discharged to rehabilitation facility, a decrease in 2.63 days in the collaborative NP group of 122 patients could yield a cost savings of US$ 641 476 per year. For the subgroup of patients transferred from another service, similar cost analysis generated a total savings of US$ 1 059 480 per year. For the subgroup of patients who are 60 years and above, similar cost analysis generated a savings of US$ 790 240 per year. For the subgroup of patients discharged on IV antibiotics or wound therapy, similar cost analysis generated savings of US$ 478 240 per year.	• A USA study conducted at single site. • Direct costs were not determined • Indirect costs in terms of dollars saved per day were computed. • Cost analysis was only done for subgroups which had significantly different LOS. • Many patients were part of more than one subgroup hence, the cost-benefit analysis could not be additive.
Scherzer et al. (2016) [41]	No significant difference in charges observed between study groups, US$ 242 324.03 ± 235 749.24 in collaborative NP group versus US$ 216 726.51 ± 262 021.77 (*t* test, *P* = 0.561).	• A USA study conducted at a single site. • Despite the longer ICU LOS in the collaborative NP group, the overall hospital charges observed was comparable to that of the resident group. • Resource utilization was similar in both groups, supporting the contention that NPs are cost-effective healthcare providers.
Skinner et el. (2013) [34]	Annual staffing cost of NP and junior residents was £933 344 with the usual model of care and £764 691 with the collaborative NP model of care.	• A UK study conducted at a single site. • A reduction of staffing costs was observed. • Uncertain of how cost analysis was done.

### Emergency setting

#### Length of stay

Four out of the 15 studies examined the impact of the advanced nursing practice roles on the length of stay in the emergency setting [28, 32, 33, 42].

##### NP-directed management of care

Two studies [28, 33] reported a significant reduction in the length of stay in EDs of patients who were reviewed and treated by NPs when compared to those seen by physicians. However, the shorter time was attributed to the baseline difference in patients’ acuity between the groups. The physicians handled patients of higher acuity and complexity than NPs. On the contrary, a multisite study [42], with comparison groups of similar baseline patient acuity, found comparable lengths of stay in EDs when patients with chest pain were managed by either NPs or physicians.

##### Length of stay in collaborative care involving nurse practitioners

One study [32] compared NP-physician collaborative model of care with usual physician-only model of care and found similar lengths of stay in ED between the comparison groups.

#### Waiting time

Of the 15 studies, six studies [28–30, 32, 33] examined the impact of advanced nursing practice roles on waiting time in the emergency setting.

##### Time to consultation

Only one study [33] reported that patients with minor injuries experienced shorter waiting time (median 14 min) when reviewed by emergency NPs than those reviewed by physicians (median 50 min). The other three studies [28, 30] comparing NP-directed care with physician-only care found similar waiting time to consultation in EDs. Another study [32] comparing the NP-physician collaborative care with physician-only care also found similar waiting time to consultation in EDs.

##### Time to treatment

One RCT [29] illustrated that a greater proportion of patients (15.4%) managed by emergency NPs received analgesia within 30 min of arrival at the ED compared to patients managed by physicians (1.6%) (*P* < 0.001).

#### Patient satisfaction

Of the 15 studies, two examined patient satisfaction in the emergency setting [30]. The two used previously validated questionnaires to measure patient satisfaction. One of which [42] found similar patient satisfaction scores when comparing NP-directed care with physician-only care while the other [30] reported NPs to receive higher patient satisfaction scores than physicians (NP median score 23 [IQR 20–24] vs. physician median score [IQR 16–24]; *P* = 0.002).

### Critical care setting

#### Length of stay

Seven out of the 15 studies examined the impact of the advanced nursing practice roles on the length of stay in the critical care setting [31, 35–38, 40, 41].

##### NP-directed management of care

Comparable lengths of stay in a trauma center was reported in one study [40] where the comparison groups had similar baseline patient acuity. A RCT [31] conducted in a post-cardiac surgery unit where patients required critical care found comparable lengths of stay in hospital between the comparison groups (NP-directed care versus physician-only care). Despite the higher acuity of care required by patients under NP-directed care than those under physician-only care, the discharge outcomes were similar. In addition, a large cohort study [38] reported a significantly shorter length of stay in medical ICUs for patients whose management were led by NPs than those under physician-only management. Patients in the NP-directed group also had lower odds (odds ratio 0.87, *P* < 0.001) of longer hospital stays. Interestingly, a higher patient-to-provider ratio was observed in the NP-directed group but the authors [38] were judicious in inferring greater efficiency in NP-directed care given the differences in the patients’ characteristics between comparison groups.

##### Collaborative care involving nurse practitioners

All included studies that compared NP-physician collaborative model of care with usual physician-only model of care found similar lengths of hospital stay [35–37, 41] between the comparison groups. However, in one study [36], after subgroup analysis, a significantly shorter length of stay was found in the physician-NP collaborative group for patients transferred from another service (mean difference 6.54 days, *P* < 0.0001), patients discharged to rehabilitation facility (mean difference 2.63 days, *P* = 0.0024), patients older than 60 years (mean difference 1.80 days, *P* = 0.0369), or patients discharged on intravenous antibiotics/wound therapy (mean difference 3.93 days, *P* = 0.0171). The management of such patients warrants greater communication with multidisciplinary teams, discharge planning, care coordination, and administrative work were required; in this niche, NPs are familiar with such tasks and can competently perform them [43].

#### Waiting time

##### Time to treatment

Only one study [39] examined the impact of advanced nursing practice roles on waiting time in the critical care setting. The study [39] demonstrated that a 24/7, on-site coverage with an acute care NP as first responders for acute ischemic stroke significantly reduced the time to treatment (median 45 min; IQR 35–58 min) in comparison to the usual service model (median 53 min; IQR 45–73 min) (*P* < 0.001).

#### Mortality

Five [34, 37–39, 41] out of the 15 studies analyzed the impact of the advanced nursing practice roles on hospital and ICU mortality. Two studies [38, 39] comparing NP-directed care with physician-only care found comparable patient mortality. One of them, a large cohort study (*n* = 9066) conducted in the medical ICU [38], suggested NP-directed care had the same quality as physician-only care. The patients under NP-directed care had lower ICU mortality (6.3%) than those under physician-only care (11.6%; adjusted OR 0.77; 95% CI 0.63–0.94; *P* = 0.01) whereas hospital mortality between groups were similar (10 vs. 15.9%; adjusted OR 0.87; 95% CI 0.73–1.03; *P* = 0.11). This finding was consistent with that in the other three studies conducted in ICUs [34, 37, 41] which compared the NP-physician collaborative care with physician-only care.

#### Patient satisfaction

Of the 15 studies, only one examined patient satisfaction in the critical care settings [31]. The study developed a new self-reported tool to measure patient satisfaction and found similar scores when comparing NP-directed care with physician-only care. Nonetheless, the study [31] reported that NPs performed better than physicians in teaching, answering questions, listening, and pain management. This finding was akin to the study [30] conducted in the ED which assessed the healthcare provider for completeness of care, politeness of service provider, explanation and advice given, waiting time, and comprehension of discharge instruction.

#### Cost

Three of the 15 studies reviewed the impact of the advanced nursing practice roles on cost [34, 36, 41], all of which compared NP-physician collaborative care with physician-only care in the critical care setting. One study [41] reported that despite a longer ICU stay for patients in the NP-physician group than for those in physician-only group, there was no significant difference in the observed charges between them. This supports the contention that involving NPs in the management of the critically ill can lead to cost savings. The other two studies [34, 36] had results that demonstrated cost savings in the NP-physician group compared to physician-only group. One of them concluded that an annual staffing cost of approximately £170 000 could be saved when physicians worked with NP in managing ICU patients.

## Discussion

With population aging and the consequent global epidemic of chronic diseases, healthcare demands will only rise. Accordingly, nurses in advanced practice can add value and increase access to healthcare by, potentially strengthening the healthcare workforce. Nonetheless, the expansion of role and autonomy of nurses will lead to concerns of patient safety and clinical outcomes. Through the narrative synthesis of the available evidence from Australia, Canada, New Zealand, UK, and USA, nurses in advanced practice appear to generate clinical outcomes comparable to those of physicians in the emergency and critical settings.

Generally, in the ICU setting, the involvement of NPs in managing the critically ill allowed for greater continuity of care [37], as NPs did not have to be on frequent rotation coverage as junior physicians. Hence, NPs developed greater familiarity with the environment and patient demands than the physicians who were constantly on rotation. The involvement of NPs also provided the unit’s staff with a consistent point of contact for the multidisciplinary team [35]. When daily multidisciplinary rounds were initiated by NPs, the coordination of care was shown to improve [40]. Providing effective care coordination is a forte of nurses [10]. Care coordination requires interpersonal communication and collaboration. As nurses can establish more personal and tangible relationships with patients than do physicians [44], they perform better in care coordination. The value of NPs was exemplified when the patient care required cross-disciplinary communication, discharge planning, follow-up care, and administrative work. With NPs’ involvement, patients’ length of stay was shortened [36]. Apart from delivering efficient care, nurses in advanced practice will get to develop expertise for managing specific groups of patients through assigned responsibilities [35].

One of the prioritized quality-of-care indicators in the emergency setting is the time from arrival to first assessment by physician [45]. This review has demonstrated that NPs were capable of rendering emergency care services as timely [28, 32] as, if not faster [33] than, physicians. The addition of nurses in advanced practice in the emergency settings enabled physicians to pay greater attention to patients of higher complexity and acuity, thereby, improving access to prompt emergency care.

Time to treatment is also a priority in emergency care. The time to first administration of analgesia is an important quality-of-care indicator in EDs [45]. There are national targets in place to improve this aspect of care. In Australia, New Zealand, and the USA, the national target for time to analgesia is 30 min from time of arrival [46, 47] and, in the UK, it is 20 min [48]. When compared with physicians, NPs were observed to have greater adherence to the recommended targets for administering analgesia in a timely fashion [29]. In their provision of a hybrid model of care amalgamating nursing and medical tasks, NPs are trained to perform patient assessment and, in some countries, have prescription rights. These factors contributed to a shortened time to treatment in the emergency setting for patients [29].

The experience of the patient is highly valued in the healthcare system [49]. This review showed that patients’ level of satisfaction was not dependent on whom but how the care was delivered [30, 31]. NPs were rated to perform better at patient education, answering queries, listening, and pain management than physicians [31]. These are the strengths of NPs, consistent with the NP goals and education, which are grounded in nursing [43, 50].

Cost savings are an important outcome measure in evaluating the feasibility of any new service model [51]. Findings from this review suggest greater cost savings with the implementation of the advanced nursing practice role in emergency or critical care [34, 36, 41]. However, judicious interpretation of the evidence is recommended. A fair synthesis of the cost savings in the included studies could not be performed as they had been done in different countries. The varying financial and funding models make it difficult to synthesize the findings. Furthermore, none of the studies in this review performed any cost-effectiveness analysis.

**Fig. 2:**
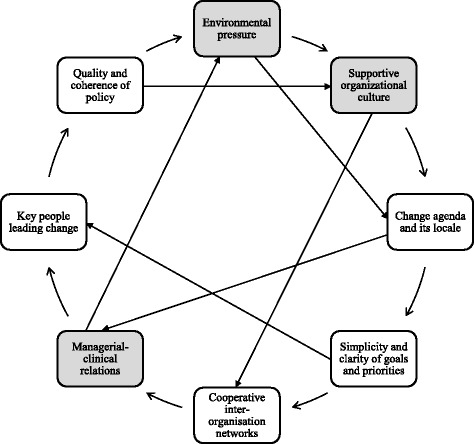
Receptive contexts for change framework

The existing evidence has demonstrated the positive impact of advanced nursing practice roles in the emergency and critical setting, it is then of benefit to examine the necessary conditions for its implementation and receptivity. According to Pettigrew et al.’s “receptive contexts for change” framework (Fig. 2), there are eight dynamically linked factors which influence the receptivity to change [52]. Three of which are especially apparent in the studies featured in this review. They are namely the presence of environmental pressure, supportive organizational culture, and managerial-clinical relations.

Environmental pressure can be especially pivotal in creating favorable conditions for change. When considering environmental pressure, besides the entire healthcare system, the political context of the country has an integral role in defining the environment [53]. Political influence, a large environmental pressure, was evident in the studies conducted in the USA [35–41] and UK [34]. In the USA, the implementation of the Affordable Care Act in 2010 was a catalyst for the development of more efficient healthcare delivery models to cope with the projected influx of new patients. In the UK study [34] featured in this review, political influence was also observed. The enactment of provisional immigration laws for physicians outside of the European Union and the European Working Time Directive has make it more difficult to support safe staff-to-patient ratios in the critical care setting. The political context of the country created an environmental pressure which consequently compelled the institutions [34–41] cited in this review to capitalize on nurses in advanced practice and experiment with new models of care delivery.

The environmental pressures trigger the development of a supportive organizational culture to effect change to ease the pressure [52]. A supportive organizational culture strives to promote staff engagement [53]. Staff engagement involves autonomy to be extended, and it was apparent in the included studies. In this review, the NPs were given greater autonomy to either practice independently [28–31, 33, 34] or collaborate [32, 35–41] with physicians at greater extents in the emergency and critical care settings. In these studies, the institutions’ willingness to take risks and evaluate new workforce utilization strategies possibly led to the successful implementation of the advanced practice nursing role [53].

Effective managerial-clinical relations is also a crucial factor in leveraging institutional change [53]. In the study conducted in Canada [31], the authors attributed the success observed in the NP role implementation in the post-operative cardiac surgery unit to the support from and collaboration between the administrators and clinical staff. As the NP role was fairly new in the study’s [31] setting then, it was necessary to involve individuals at all levels in the NP role implementation to optimize its success [54]. One approach to facilitate effective managerial-clinical relations is through adopting a distributed model of leadership [55], which encourages collaboration between the administrators and clinical staff. The distributed leadership approach is known to be most efficacious where job roles are mutually dependent [56]. The implementation of advanced practice nursing roles in the emergency and critical care settings involves mutually dependent job roles and so will benefit from the distributed leadership approach. The distributed leadership approach utilizes a bottom-up process, where individuals working in the setting-of-interest participates in decision-making [55]. Using this approach creates the notion of co-construction, which avoids the overreliance on a dominant individual, increasing the likelihood for sustainable change [54].

The quality and coherence of policy is one factor in the receptive context framework [52] which was not discussed in the included studies but is vital in the implementation of the NP/APN role. The lack of coherent policy to define the roles and professional boundaries of advanced nursing practice can cause healthcare administrators to be apprehensive about the implementation of healthcare models where NP/APNs are given more autonomy and responsibilities [57–59]. State law governs advanced nursing practice and define supervisory requirements [60]. Often, the legal frameworks lack clarity on the legal accountability of physicians, should nurses under the physicians’ supervision commit errors harmful to patients [61, 62]. Professional indemnity is closely associated to legislative boundaries [63]. The successful implementation of the NP/APN role hinges on the institution of relevant regulatory frameworks and credentialing systems to guide policy implementations and educational establishments [64]. It, therefore, reiterates the importance of having coherent policies to define roles and professional independence of nurses in advanced practice.

### Limitations

The meta-analysis of the outcomes was not done to present the combined effect of estimates on the impact of advanced nursing roles in the emergency and critical care settings. Yet, to perform a meta-analysis would be inappropriate as the included studies were heterogeneous in designs, interventions, and outcome measures. The heterogeneity of studies was expected as the professional boundaries of nurses differ across countries. However, a review of the impact of advance nursing practice across countries is still valuable.

A limitation in all studies is the poor definition and description of the scope of advanced nursing practice. In addition, preparatory training for nurses to assume advanced practice was rarely discussed. The level of theoretical knowledge and clinical competence of the nurses might differ across the studies; hence, the comparison might not have been fair.

Finally, despite the search across nine international databases, this review included papers in only English; relevant papers not published in English might have been omitted.

## Conclusion

Capitalizing on nurses in advanced practice to increase patients’ access to emergency and critical care is appealing and beneficial. This review suggests that the implementation of the NP/APN role in the emergency and critical care settings improves patient outcomes. The transformation of healthcare delivery through effective utilization of the workforce may alleviate the impending rise in demand for health services. Nevertheless, it is necessary to first prepare a receptive context to effect sustainable change.

## Additional files

Additional file 1: Search strategy.

Additional file 2: List of studies excluded after critical appraisal.

## Abbreviations

APN: Advanced practice nurses
ED: Emergency department
ICU: Intensive care unit
JBI: Joanna Briggs Institute
NP: Nurse practitioners
PICO: Population, Intervention, Comparison, and Outcome
PRISMA: Preferred Reporting Items for Systematic Reviews and Meta-Analyses
RCT: Randomized controlled trial
UK: United Kingdom
USA: United States of America
