# Supplementary material for: The impact of the advanced practice nursing role on quality of care, clinical outcomes, patient satisfaction, and cost in the emergency and critical care settings: a systematic review
Source: Hum Resour Health. 2017 Sep 11;15:63. doi: 10.1186/s12960-017-0237-9 (PMC5594520; doi:10.1186/s12960-017-0237-9)
Supplement: Supplementary file 1 — Search strategy. [file 12960_2017_237_MOESM1_ESM.docx]

**Additional file 1:** Search strategy

| **Database** | **Keywords** |
| --- | --- |
| **PubMed** | **SEARCH #1**  (("Nurse practitioner*"[Title/Abstract] OR "Nurse clinician*"[Title/Abstract] OR "Non-physician"[Title/Abstract] OR "Advanced practice nurse"[Title/Abstract] OR "Advanced nursing practice"[Title/Abstract])) OR ("Nurse Practitioners" OR "Nurse Clinicians" OR "Nursing Staff" OR "Advanced Practice Nursing"[MeSH Terms])  **AND**  (("Patient management"[Title/Abstract] OR "Patient outcome"[Title/Abstract] OR "Treatment Outcome"[Title/Abstract] OR "Patient satisfaction"[Title/Abstract] OR "Hospitali*"[Title/Abstract] OR "Patient Readmission"[Title/Abstract] OR "Mortality"[Title/Abstract] OR "Hospital Cost*"[Title/Abstract] OR "Clinical Competence"[Title/Abstract] OR "Survival"[Title/Abstract] OR "Time Factor*"[Title/Abstract] OR "Staffing*"[Title/Abstract] OR "Schedul*"[Title/Abstract] OR "Workload"[Title/Abstract] OR "Efficiency"[Title/Abstract] OR "Length of stay"[Title/Abstract] OR "Wait* time"[Title/Abstract] OR "Complication rate*"[Title/Abstract] OR "Complication*"[Title/Abstract] OR "Quality of care"[Title/Abstract] OR "Cost* of care"[Title/Abstract] OR "Job satisfaction"[Title/Abstract] OR "Staff perception" [Title/Abstract] OR "Staff satisfaction"[Title/Abstract])) OR ("Outcome and Process Assessment (Health Care)" OR "Cost-Benefit Analysis" OR "Patient Satisfaction" OR "Treatment Outcome" OR "Cost Savings" OR "Critical Care Outcomes" OR "Hospital Mortality" OR "Hospital Costs" OR "Social Perception" OR "Hospitalization" OR "Patient Readmission" OR "Health Services Research" OR "Quality of Health Care" OR "Utilization Review" OR "Referral and Consultation" OR "Clinical Competence" OR "Feasibility Studies" OR "Job Description" OR "Program Development" OR "Nursing Evaluation Research" OR "Program Evaluation" OR "Survival Analysis" OR "Professional Role" OR "Evidence-based Medicine" OR "Time Factors" OR "Practice Guidelines as Topic" OR "Models, Nursing” OR “Personnel Staffing and Scheduling" OR "Workload" OR "Efficiency, Organizational" OR "Hospitals, University/manpower"[MeSH Terms])  **AND**  ((“Intensive care unit”[Title/Abstract] OR “Intensive care”[Title/Abstract] OR “Critical care unit”[Title/Abstract] OR “Critically ill*”[Title/Abstract] OR “Subacute care”[Title/Abstract] OR “High dependency care”[Title/Abstract] OR “High dependency unit”[Title/Abstract] OR “Emergency”[Title/Abstract] OR “Acute care”[Title/Abstract] OR “Acute disease”[Title/Abstract] OR “Acute illness”[Title/Abstract] OR “Trauma”[Title/Abstract] OR “Post-op*”[Title/Abstract])) OR (“Intensive Care Units” OR “Emergency Medical Services” OR “Acute Disease” OR “Emergencies” OR “Heart Arrest” OR “Emergency Service, Hospital” OR “Resuscitation” OR “Subacute Care” OR “Critical Care” OR “Critical Illness” OR “Post-operative Care” OR “Critical Pathways”[MeSH Terms])  **SEARCH #2**  (("Nurse practitioner*"[Title/Abstract] OR "Nurse clinician*"[Title/Abstract] OR "Non-physician"[Title/Abstract] OR " Advanced practice nurse"[Title/Abstract] OR "Advanced nursing practice"[Title/Abstract])) OR ("Nurse Practitioners" OR "Nurse Clinicians" OR "Nursing Staff" OR "Advanced Practice Nursing"[MeSH Terms])  **AND**  ((“Physician*”[Title/Abstract] OR “Doctor*”[Title/Abstract] OR “Medical practitioner*”[Title/Abstract] OR “Interdisciplin*”[Title/Abstract] OR “Case manage*”[Title/Abstract] OR “Cooperative behaviour”[Title/Abstract] OR “Cooperative behavior”[Title/Abstract] OR “Physician-Nurse”[Title/Abstract])) OR (“Patient Care Team” OR “Medical Staff, Hospital” OR “Case management” OR “Physician Executives” OR “Organizational Innovation” OR “Physician-Nurse Relations” OR “Models, Organizational” OR “Hospital, Teaching” OR “Cooperative Behavior” OR “Physicians” OR “Interdisciplinary Communication”[MeSH Terms])  **AND**  ((“Intensive care unit”[Title/Abstract] OR “Intensive care”[Title/Abstract] OR “Critical care unit”[Title/Abstract] OR “Critically ill*”[Title/Abstract] OR “Subacute care”[Title/Abstract] OR “High dependency care”[Title/Abstract] OR “High dependency unit”[Title/Abstract] OR “Emergency”[Title/Abstract] OR “Acute care”[Title/Abstract] OR “Acute disease”[Title/Abstract] OR “Acute illness”[Title/Abstract] OR “Trauma”[Title/Abstract] OR “Post-op*”[Title/Abstract])) OR (“Intensive Care Units” OR “Emergency Medical Services” OR “Acute Disease” OR “Emergencies” OR “Heart Arrest” OR “Emergency Service, Hospital” OR “Resuscitation” OR “Subacute Care” OR “Critical Care” OR “Critical Illness” OR “Post-operative Care” OR “Critical Pathways”[MeSH Terms]) |
| **CINAHL** | **SEARCH #1**  AB (“Nurse practitioner*” OR “Nurse clinician*” OR “Non-physician” OR “Advance* practice nurs*” OR “Advance* Nurs* pract*”) OR MM (“Nursing Staff, Hospital” OR "Advanced Practice Nurses+" OR "Advanced Nursing Practice+")  **AND**  AB (“Patient management” OR “Patient outcome” OR “Treatment Outcome” OR “Patient satisfaction” OR “Hospitali?ation” OR “Patient Readmission” OR “Mortality” OR “Hospital Cost*” OR “Clinical Competence” OR “Survival” OR “Time Factor*” OR “Staffing*” OR “Schedul*” OR “Workload” OR “Efficienc*” OR “Length of stay” OR “Wait* time” OR “Complication rate*” OR “Complication*” OR “Quality of care” OR “Cost* of care” OR “Job satisfaction” OR “Staff perception” OR “Staff satisfaction” OR “Social perception” ) OR MM ( “Outcome Assessment” OR “Cost Benefit Analysis” OR “Patient Satisfaction” OR “Treatment Outcomes+” OR “Cost Savings” OR “Hospital Mortality” OR “Health Care Costs+” OR “Hospitalization+” OR “Readmission” OR “Length of Stay” OR “Health Services Research+” OR “Quality of Health Care+” OR “Utilization Review” OR “Referral and Consultation+” OR “Clinical Competence+” OR “Job Description” OR “Program Development+” OR “Survival Analysis+” OR “Nursing Role” OR “Role Change” OR “Time Factors” OR “Practice Guidelines” OR “Nursing Models, Theoretical” OR “Personnel Staffing and Scheduling” OR “Personnel Shortage” OR “Workload” OR “Organizational Efficiency+”)  **AND**  AB (“Intensive care unit” OR “Intensive care” OR “Critical care unit” OR “Critically ill*” OR “Subacute care” OR “High dependency care” OR “High dependency unit” OR “Emergency” OR “Acute care” OR “Acute disease” OR “Acute illness” OR “Trauma” OR “Post-operat*” OR “Postoperat*” ) OR MM ( “Intensive Care Units+” OR “Emergency Medical Services +” OR “Acute Disease” OR “Heart Arrest+” OR “Emergency Service+” OR “Resuscitation+” OR “Subacute Care” OR “Critical Care” OR “Critical Illness” OR “Critically Ill Patients” OR “Emergency Patients” OR “Postoperative Care” OR “Post Anesthesia Care”)  **SEARCH #2**  AB (“Nurse practitioner*” OR “Nurse clinician*” OR “Non-physician” OR “Advance* practice nurs*” OR “Advance* Nurs* pract*”) OR MM (“Nursing Staff, Hospital” OR "Advanced Practice Nurses+" OR "Advanced Nursing Practice+")  **AND**  AB (“Physician*” OR “Doctor*” OR “Medical practitioner*” OR “Interdisciplin*” OR “Case manage*” OR “Cooperative behaviour” OR “Cooperative behavior” OR “Physician-Nurse”) OR MM (“Multidisciplinary Care Team” OR “Medical Staff+” OR “Case management” OR “Physician Executives” OR “Interprofessional relations+” OR “Cooperative Behavior” OR “Physicians+”)  **AND**  AB ( “Intensive care unit” OR “Intensive care” OR “Critical care unit” OR “Critically ill*” OR “Subacute care” OR “High dependency care” OR “High dependency unit” OR “Emergency” OR “Acute care” OR “Acute disease” OR “Acute illness” OR “Trauma” OR “Post-operat*” OR “Postoperat*” ) OR MM ( “Intensive Care Units+” OR “Emergency Medical Services +” OR “Acute Disease” OR “Heart Arrest+” OR “Emergency Service+” OR “Resuscitation+” OR “Subacute Care” OR “Critical Care” OR “Critical Illness” OR “Critically Ill Patients” OR “Emergency Patients” OR “Postoperative Care” OR “Post Anesthesia Care” ) |
| **The Cochrane Library** | **SEARCH #1**  ("Nurse practitioner*" OR "Nurse clinician*" OR "Non-physician" OR "Advance* practice nurs*" OR "Advance* Nurs* pract*"):ab  OR MeSH descriptor ("Nurse Practitioners" OR "Nurse Clinicians" OR "Nursing Staff" OR "Advanced Practice Nursing") explode all trees  **AND**  ("Patient management" OR "Patient outcome" or "Treatment Outcome" OR "Patient satisfaction" OR "Hospitali?ation" OR "Patient Readmission" OR "Mortality" OR "Hospital Cost*" OR "Clinical Competence" OR "Survival" OR "Time Factor*" OR "Staffing*" OR "Schedul*" OR "Workload" OR "Efficienc*" OR "Length of stay" OR "Wait* time" OR "Complication rate*" OR "Complication*" OR "Quality of care" OR "Cost* of care" OR "Job satisfaction" OR "Staff perception" OR "Staff satisfaction" or "Social perception": )ab OR MeSH descriptor ("Outcome and Process Assessment (Health Care)" OR "Cost-Benefit Analysis" OR "Patient Satisfaction" OR "Treatment Outcome" OR "Cost Savings" OR "Critical Care Outcomes" OR "Hospital Mortality" OR "Hospital Costs" OR "Social Perception" OR "Hospitalization" OR "Patient Readmission" OR "Health Services Research" OR "Quality of Health Care" OR "Utilization Review" OR "Referral and Consultation" OR "Clinical Competence" OR "Feasibility Studies" OR "Job Description" OR "Program Development" OR "Nursing Evaluation Research" OR "Program Evaluation" OR "Survival Analysis" OR "Professional Role" OR "Evidence-based Medicine" OR "Time Factors" OR "Practice Guidelines as Topic" OR "Models, Nursing” OR “Personnel Staffing and Scheduling" OR "Workload" OR "Efficiency, Organizational”) explode all trees  **AND**  ("Intensive care unit" or "Intensive care" or "Critical care unit" or "Critically ill*" or "Subacute care" or "High dependency care" or "High dependency unit" or "Emergency" or "Acute care" or "Acute disease" or "Acute illness" or "Trauma" or "Post-operat*" or "Postoperat*"):ab OR MeSH descriptor (“Intensive Care Units” OR “Emergency Medical Services” OR “Acute Disease” OR “Emergencies” OR “Heart Arrest” OR “Emergency Service, Hospital” OR “Resuscitation” OR “Subacute Care” OR “Critical Care” OR “Critical Illness” OR “Postoperative Care” OR “Critical Pathways”) explode all trees  **SEARCH #2**  ("Nurse practitioner*" OR "Nurse clinician*" OR "Non-physician" OR "Advance* practice nurs*" OR "Advance* Nurs* pract*"):ab  OR MeSH descriptor ("Nurse Practitioners" OR "Nurse Clinicians" OR "Nursing Staff" OR "Advanced Practice Nursing") explode all trees  **AND**  “"Physician*" OR "Doctor*" OR "Medical practitioner*" OR "Interdisciplin*" OR "Case manage*" OR "Cooperative behaviour" OR "Cooperative behavior" OR "Physician-Nurse"):ab OR MeSH descriptor (“Patient Care Team” OR “Medical Staff, Hospital” OR “Case management” OR “Physician Executives” OR “Organizational Innovation” OR “Physician-Nurse Relations” OR “Models, Organizational” OR “Hospital, Teaching” OR “Cooperative Behavior” OR “Physicians” OR “Interdisciplinary Communication”) explode all trees  **AND**  ("Intensive care unit" or "Intensive care" or "Critical care unit" or "Critically ill*" or "Subacute care" or "High dependency care" or "High dependency unit" or "Emergency" or "Acute care" or "Acute disease" or "Acute illness" or "Trauma" or "Post-operat*" or "Postoperat*"):ab OR MeSH descriptor (“Intensive Care Units” OR “Emergency Medical Services” OR “Acute Disease” OR “Emergencies” OR “Heart Arrest” OR “Emergency Service, Hospital” OR “Resuscitation” OR “Subacute Care” OR “Critical Care” OR “Critical Illness” OR “Postoperative Care” OR “Critical Pathways”) explode all trees |
| **Scopus** | **SEARCH #1**  TITLE-ABS-KEY ("Nurse practitioner*" OR "Nurse clinician*" OR "Non-physician" OR "Advance* practice nurs*" OR "Advance* Nurs* pract*")  **AND**  TITLE-ABS-KEY ("Patient management" OR "Patient outcome" OR "Treatment Outcome" OR "Patient satisfaction" OR "Hospitali*" OR "Patient Readmission" OR "Mortality" OR "Hospital Cost*" OR "Clinical Competence" OR "Survival" OR "Time Factor*" OR "Staffing*" OR "Schedul*" OR "Workload" OR "Efficienc*" OR "Length of stay" OR "Wait* time" OR "Complication rate*" OR "Complication*" OR "Quality of care" OR "Cost* of care" OR "Job satisfaction" OR "Staff perception" OR "Staff satisfaction" OR "Social perception" OR "Cost-Benefit" OR "Cost Saving*" OR "Critical Care Outcome*" OR "Hospital Mortality" OR "Hospital Cost*" OR "Hospitali?ation" OR "Patient Readmi*" OR "Clinical Competenc*" OR "Feasibility Stud*" OR "Job Description" OR "Program Development" OR "Nursing Evaluat*" OR "Program Evalua*" OR "Professional Role" OR  "Practice Guideline*" OR "Nursing Model" OR "Model of Nursing" OR "Workload" OR "Manpower")  **AND**  TITLE-ABS-KEY ("Intensive care unit*" OR "Intensive care" OR "Critical care unit*" OR "Critical* ill*" OR "Subacute care" OR "High dependency care" OR "High dependency unit*" OR "Emergenc*" OR "Acute care" OR "Acute disease*" OR "Acute illness*" OR "Trauma" OR "Post-operat*" OR "Postoperat*")  **SEARCH #2**  TITLE-ABS-KEY ("Nurse practitioner*" OR "Nurse clinician*" OR "Non-physician" OR "Advance* practice nurs*" OR "Advance* Nurs* pract*")  **AND**  TITLE-ABS-KEY ("Physician*" OR "Doctor*" OR "Medical practitioner*" OR "Interdisciplin*" OR "Case manage*" OR "Cooperative behav*" OR "Physician-Nurse" OR “Doctor-Nurse” OR "Patient Care Team" OR "Medical Staff" OR "Physician Executive*" OR "Organi?ational Innovation*" OR "Organi?ational model*")  **AND**  TITLE-ABS-KEY ("Intensive care unit" OR "Intensive care" OR "Critical care unit" OR "Critically ill*" OR "Subacute care" OR "High dependency care" OR "High dependency unit" OR "Emergency" OR "Acute care" OR "Acute disease" OR "Acute illness" OR "Trauma" OR "Post-operat*" OR "Postoperat*") |
| **Embase** | **SEARCH #1**  'nursing staff'/de OR 'nurse practitioner*':ab,ti OR 'nurse clinician*':ab,ti OR 'non-physician':ab,ti OR 'advance* practice nurs*':ab,ti OR 'advance* nurs* pract*':ab,ti OR 'advanced practice nurse'/exp OR 'advanced practice nursing'/exp  **AND**  'patient management':ab,ti OR 'patient outcome':ab,ti OR 'treatment outcome':ab,ti OR 'patient satisfaction':ab,ti OR 'hospitalisation':ab,ti OR 'hospitalization':ab,ti OR 'patient readmission':ab,ti OR 'mortality':ab,ti OR 'hospital cost*':ab,ti OR 'clinical competence':ab,ti OR 'survival':ab,ti OR 'time factor*':ab,ti OR 'staffing*':ab,ti OR 'schedul*':ab,ti OR 'workload':ab,ti OR 'efficienc*':ab,ti OR 'length of stay':ab,ti OR 'wait* time':ab,ti OR 'complication rate*':ab,ti OR 'complication*':ab,ti OR 'quality of care':ab,ti OR 'cost* of care':ab,ti OR 'job satisfaction':ab,ti OR 'staff perception':ab,ti OR 'staff satisfaction':ab,ti OR 'social perception':ab,ti OR “cost benefit analysis”/de OR “patient satisfaction”/de OR “hospital readmission”/de OR “health services research”/de OR “utilization review”/de OR “patient referral”/de OR “feasibility study”/de OR “job satisfaction”/de OR “program development”/de OR “nursing evaluation research”/de OR “survival rate”/de OR “survival prediction”/de OR “scope of practice”/de OR “turnaround time”/de OR “workload”/de OR “organizational efficiency”/de OR “[health](http://www-ncbi-nlm-nih-gov.libproxy1.nus.edu.sg/pubmed/?term=Improved+quality+of+care+and+reduction+of+housestaff+workload+using+trauma+nurse+practitioners.) care manpower”/de OR “mortality”/exp OR “hospital Cost”/exp OR “hospitalization”/exp OR “health care quality”/exp OR “competence”/exp OR “program evaluation”/exp OR “evidence based medicine”/exp OR “personnel management”/exp  **AND**  ‘Intensive care unit*’:ab,ti OR ‘intensive care’:ab,ti OR ‘critical care unit*’:ab,ti OR ‘critically ill*’:ab,ti OR ‘subacute care’:ab,ti OR ‘high dependency care’:ab,ti OR ‘high dependency unit’:ab,ti OR ‘emergenc*’:ab,ti OR ‘acute care’:ab,ti OR ‘acute disease*’:ab,ti OR ‘acute illness*’:ab,ti OR ‘trauma’:ab,ti OR ‘post-operat*’:ab,ti OR ‘postoperat*’:ab,ti OR ‘intensive care unit’/de OR ‘emergency health service’/de OR ‘acute disease’/de OR ‘emergency’/de OR ‘critical illness’/de OR ‘clinical pathway’/de OR ‘heart arrest’/exp OR ‘intensive care’/exp OR ‘postoperative period’/exp  **SEARCH #2**  'nursing staff'/de OR 'nurse practitioner*':ab,ti OR 'nurse clinician*':ab,ti OR 'non-physician':ab,ti OR 'advance* practice nurs*':ab,ti OR 'advance* nurs* pract*':ab,ti OR 'advanced practice nurse'/exp OR 'advanced practice nursing'/exp  **AND**  ‘physician*’:ab,ti OR ‘doctor*’:ab,ti OR ‘medical practitioner*’:ab,ti OR ‘interdisciplin*’:ab,ti OR ‘case manage*’:ab,ti OR ‘cooperative behav*’:ab,ti OR ‘physician-nurse’:ab,ti OR ‘medical staff’/de OR ‘case management’/de OR ‘organizational efficiency’/de OR ‘doctor nurse relation’/de OR ‘interdisciplinary communication’/de OR ‘teaching hospital’/exp OR ‘cooperation’/exp OR ‘physician’/exp  **AND**  ‘Intensive care unit*’:ab,ti OR ‘intensive care’:ab,ti OR ‘critical care unit*’:ab,ti OR ‘critically ill*’:ab,ti OR ‘subacute care’:ab,ti OR ‘high dependency care’:ab,ti OR ‘high dependency unit’:ab,ti OR ‘emergenc*’:ab,ti OR ‘acute care’:ab,ti OR ‘acute disease*’:ab,ti OR ‘acute illness*’:ab,ti OR ‘trauma’:ab,ti OR ‘post-operat*’:ab,ti OR ‘postoperat*’:ab,ti OR ‘intensive care unit’/de OR ‘emergency health service’/de OR ‘acute disease’/de OR ‘emergency’/de OR ‘critical illness’/de OR ‘clinical pathway’/de OR ‘heart arrest’/exp OR ‘intensive care’/exp OR ‘postoperative period’/exp |
| **Web of Science** | **SEARCH #1**  TOPIC: ((Nurse practitioner*) OR (Nurse clinician*) OR (Non-physician*) OR (Nonphysician*) OR (Advance* practice nurs*) OR (Advance* Nurs* pract*))  **AND**  TOPIC: ((Patient management) OR (Patient outcome*) OR (Treatment Outcome*) OR (Patient satisfaction) OR (Hospitali?ation) OR (Patient Readmission) OR (Mortality) OR (Hospital Cost*) OR (Clinical Competence) OR (Survival) OR (Time Factor*) OR (Staffing*) OR (Schedul*) OR (Workload) OR (Efficienc*) OR (Length of stay) OR (Wait* time) OR (Complication rate*) OR (Complication*) OR (Quality of care) OR (Cost* of care) OR (Job satisfaction) OR (Staff perception*) OR (Staff satisfaction) OR (Social perception*) OR (Cost-Benefit*) OR (Cost Saving*) OR (Critical Care Outcome*) OR (Hospital Mortality) OR (Hospital Cost*) OR (Hospitali?ation) OR (Patient Readmi*) OR (Clinical Competenc*) OR (Feasibility Stud*) OR (Job Description) OR (Program Development) OR (Nursing Evaluat*) OR (Program Evalua*) OR (Professional Role) OR (Practice Guideline*)  OR (Nursing Model) OR (Model* of Nursing) OR (Workload) OR (Manpower))  **AND**  TOPIC: ((Intensive care unit*) OR (Intensive care) OR (Critical care unit*) OR (Critical* ill*) OR (Subacute care) OR (High dependency care) OR (High dependency unit) OR (Emergenc*) OR (Acute care) OR (Acute disease) OR (Acute illness*) OR (Trauma) OR (Post-operat*) OR (Postoperat*))  **SEARCH #2**  TOPIC: ((Nurse practitioner*) OR (Nurse clinician*) OR (Non-physician*) OR (Nonphysician*) OR (Advance* practice nurs*) OR (Advance* Nurs* pract*))  **AND**  TOPIC: ((Physician*) OR (Doctor*) OR (Medical practitioner) OR (Interdisciplin*) OR (Case manage*) OR (Cooperative behav*) OR (Physician-Nurse) OR (Doctor-Nurse) OR (Patient Care Team) OR (Medical Staff) OR (Physician Executive*) OR (Organi?ational Innovation*) OR (Organi?ational model*))  **AND**  TOPIC: ((Intensive care unit*) OR (Intensive care) OR (Critical care unit*) OR (Critical* ill*) OR (Subacute care) OR (High dependency care) OR (High dependency unit) OR (Emergenc*) OR (Acute care) OR (Acute disease) OR (Acute illness*) OR (Trauma) OR (Post-operat*) OR (Postoperat*)) |
| **ScienceDirect** | **SEARCH #1**  TITLE-ABSTR-KEY("Nurse practitioner*" OR "Nurse clinician*" OR "Non-physician" OR "Advance* practice nurs*" OR "Advance* Nurs* pract*")  **AND**  TITLE-ABSTR-KEY("Patient management" OR "Patient outcome" OR "Treatment Outcome" OR "Patient satisfaction" OR "Hospitali*" OR "Patient Readmission" OR "Mortality" OR "Hospital Cost*" OR "Clinical Competence" OR "Survival" OR "Time Factor*" OR "Staffing*" OR "Schedul*" OR "Workload" OR "Efficienc*" OR "Length of stay" OR "Wait* time" OR "Complication rate*" OR "Complication*" OR "Quality of care" OR "Cost* of care" OR "Job satisfaction" OR "Staff perception" OR "Staff satisfaction" OR "Social perception" OR "Cost-Benefit" OR "Cost Saving*" OR "Critical Care Outcome*" OR "Hospital Mortality" OR "Hospital Cost*" OR "Hospitali?ation" OR "Patient Readmi*" OR "Clinical Competenc*" OR "Feasibility Stud*" OR "Job Description" OR "Program Development" OR "Nursing Evaluat*" OR "Program Evalua*" OR "Professional Role" OR  "Practice Guideline*" OR "Nursing Model" OR "Model of Nursing" OR "Workload" OR "Manpower")  **AND**  TITLE-ABSTR-KEY("Intensive care unit*" OR "Intensive care" OR "Critical care unit*" OR "Critical* ill*" OR "Subacute care" OR "High dependency care" OR "High dependency unit*" OR "Emergenc*" OR "Acute care" OR "Acute disease*" OR "Acute illness*" OR "Trauma" OR "Post-operat*" OR "Postoperat*")  **SEARCH #2**  TITLE-ABSTR-KEY("Nurse practitioner*" OR "Nurse clinician*" OR "Non-physician" OR "Advance* practice nurs*" OR "Advance* Nurs* pract*")  **AND**  TITLE-ABSTR-KEY("Physician*" OR "Doctor*" OR "Medical practitioner*" OR "Interdisciplin*" OR "Case manage*" OR "Cooperative behav*" OR "Physician-Nurse" OR “Doctor-Nurse” OR "Patient Care Team" OR "Medical Staff" OR "Physician-Executive*" OR "Organi?ational Innovation*")  **AND**  TITLE-ABSTR-KEY("Intensive care unit*" OR "Intensive care" OR "Critical care unit*" OR "Critical* ill*" OR "Subacute care" OR "High dependency care" OR "High dependency unit*" OR "Emergenc*" OR "Acute care" OR "Acute disease*" OR "Acute illness*" OR "Trauma" OR "Post-operat*" OR "Postoperat*") |
| **Wiley Online Library** | **SEARCH #1**  "Nurse practitioner*" OR "Nurse clinician*" OR "Non-physician" OR "Advance* practice nurs*" OR "Advance* Nurs* pract*" in Abstract  **AND**  "Patient management" OR "Patient outcome" OR "Treatment Outcome" OR "Patient satisfaction" OR "Hospitali*" OR "Patient Readmission" OR "Mortality" OR "Hospital Cost*" OR "Clinical Competence" OR "Survival" OR "Time Factor*" OR "Staffing*" OR "Schedul*" OR "Workload" OR "Efficienc*" OR "Length of stay" OR "Wait* time" OR "Complication rate*" OR "Complication*" OR "Quality of care" OR "Cost* of care" OR "Job satisfaction" OR "Staff perception" OR "Staff satisfaction" OR "Social perception" OR "Cost-Benefit" OR "Cost Saving*" OR "Critical Care Outcome*" OR "Hospital Mortality" OR "Hospital Cost*" OR "Hospitali?ation" OR "Patient Readmi*" OR "Clinical Competenc*" OR "Feasibility Stud*" OR "Job Description" OR "Program Development" OR "Nursing Evaluat*" OR "Program Evalua*" OR "Professional Role" OR "Practice Guideline*" OR "Nursing Model" OR "Model of Nursing" OR "Workload" OR "Manpower" in Abstract  **AND**  "Intensive care unit*" OR "Intensive care" OR "Critical care unit*" OR "Critical* ill*" OR "Subacute care" OR "High dependency care" OR "High dependency unit*" OR "Emergenc*" OR "Acute care" OR "Acute disease*" OR "Acute illness*" OR "Trauma" OR "Post-operat*" OR "Postoperat*" in Abstract  **SEARCH #2**  "Nurse practitioner*" OR "Nurse clinician*" OR "Non-physician" OR "Advance* practice nurs*" OR "Advance* Nurs* pract*" in Abstract  **AND**  "Physician*" OR "Doctor*" OR "Medical practitioner*" OR "Interdisciplin*" OR "Case manage*" OR "Cooperative behav*" OR "Physician-Nurse" OR “Doctor-Nurse” OR "Patient Care Team" OR "Medical Staff" OR "Physician-Executive*" OR "Organi?ational Innovation*" in Abstract  **AND**  "Intensive care unit*" OR "Intensive care" OR "Critical care unit*" OR "Critical* ill*" OR "Subacute care" OR "High dependency care" OR "High dependency unit*" OR "Emergenc*" OR "Acute care" OR "Acute disease*" OR "Acute illness*" OR "Trauma" OR "Post-operat*" OR "Postoperat*" in Abstract |
| **ProQuest Dissertations & Theses Global** | **SEARCH #1**  AB,TI((Nurse practitioner*) OR (Nurse clinician*) OR (Non-physician*) OR (Nonphysician*) OR (Advance* practice nurs*) OR (Advance* Nurs* pract*))  **AND**  AB,TI((Patient management) OR (Patient outcome*) OR (Treatment Outcome*) OR (Patient satisfaction) OR (Hospitali?ation) OR (Patient preadmission) OR (Mortality) OR (Hospital Cost*) OR (Clinical Competence) OR (Survival) OR (Time Factor*) OR (Staffing*) OR (Schedul*) OR (Workload) OR (Efficienc*) OR (Length of stay) OR (Wait* time) OR (Complication rate*) OR (Complication*) OR (Quality of care) OR (Cost* of care) OR (Job satisfaction) OR (Staff perception*) OR (Staff satisfaction) OR (Social perception*) OR (Cost-Benefit*) OR (Cost Saving*) OR (Critical Care Outcome*) OR (Hospital Mortality) OR (Hospital Cost*) OR (Hospitali?ation) OR (Patient Readmi*) OR (Clinical Competenc*) OR (Feasibility Stud*) OR (Job Description) OR (Program Development) OR (Nursing Evaluat*) OR (Program Evalua*) OR (Professional Role) OR (Practice Guideline*) OR (Nursing Model) OR (Model* of Nursing) OR (Workload) OR (Manpower))  **AND**  AB,TI((Intensive care unit*) OR (Intensive care) OR (Critical care unit*) OR (Critical* ill*) OR (Subacute care) OR (High dependency care) OR (High dependency unit) OR (Emergenc*) OR (Acute care) OR (Acute disease) OR (Acute illness*) OR (Trauma) OR (Post-operat*) OR (Postoperat*))  **SEARCH #2**  AB,TI((Nurse practitioner*) OR (Nurse clinician*) OR (Non-physician*) OR (Nonphysician*) OR (Advance* practice nurs*) OR (Advance* Nurs* pract*))  **AND**  AB,TI((Physician*) OR (Doctor*) OR (Medical practitioner) OR (Interdisciplin*) OR (Case manage*) OR (Cooperative behav*) OR (Physician-Nurse) OR (Doctor-Nurse) OR (Patient Care Team) OR (Medical Staff) OR (Physician Executive*) OR (Organi?ational Innovation*) OR (Organi?ational model*))  **AND**  AB,TI((Intensive care unit*) OR (Intensive care) OR (Critical care unit*) OR (Critical* ill*) OR (Subacute care) OR (High dependency care) OR (High dependency unit) OR (Emergenc*) OR (Acute care) OR (Acute disease) OR (Acute illness*) OR (Trauma) OR (Post-operat*) OR (Postoperat*)) |
