# Supplementary material for: The impact of the advanced practice nursing role on quality of care, clinical outcomes, patient satisfaction, and cost in the emergency and critical care settings: a systematic review
Source: Hum Resour Health. 2017 Sep 11;15:63. doi: 10.1186/s12960-017-0237-9 (PMC5594520; doi:10.1186/s12960-017-0237-9)
Supplement: Supplementary file 2 — List of studies excluded after critical appraisal. [file 12960_2017_237_MOESM2_ESM.docx]

**Additional file 2:** List of studies excluded after critical appraisal

1. Considine J, Martin R, Smit D, Winter C, Jenkins J: Emergency nurse practitioner care and emergency department patient flow: Case-control study. *EMA - Emergency Medicine Australasia* 2006, 18:385-390.
2. Bevis LC, Berg-Copas GM, Thomas BW, Vasquez DG, Wetta-Hall R, Brake D, Lucas E, Toumeh K, Harrison P: Outcomes of tube thoracostomies performed by advanced practice providers vs trauma surgeons. *Am J Crit Care* 2008, 17:357-363.
3. Jarrett LA, Emmett M: Utilizing trauma nurse practitioners to decrease length of stay. *J Trauma Nurs* 2009, 16:68-72.
4. Yacopetti N, Alexandrou E, Spencer TR, Frost SA, Davidson PM, O'Sullivan G, Hillman KM: Central venous catheter insertion by a clinical nurse consultant or anaesthetic medical staff: a single-centre observational study. *Crit Care Resusc* 2010, 12:90-95.
5. Matsushima K, Inaba K, Skiada D, Esparza M, Cho J, Lee T, Strumwasser A, Magee G, Grabo D, Lam L, et al: A high-volume trauma intensive care unit can be successfully staffed by advanced practitioners at night. *Journal of Critical Care* 2016, 33:4-7.
